# Interference With Complex IV as a Model of Age-Related Decline in Synaptic Connectivity

**DOI:** 10.3389/fnmol.2020.00043

**Published:** 2020-03-24

**Authors:** Martin Kriebel, Julia Ebel, Florian Battke, Stefan Griesbach, Hansjürgen Volkmer

**Affiliations:** ^1^Department of Molecular Biology and Neurobiology, NMI Natural and Medical Sciences Institute at the University of Tübingen, Reutlingen, Germany; ^2^CeGaT GmbH, Tübingen, Germany

**Keywords:** cytochrome c oxidase, Cox4, mitochondria, aging, synaptic connectivity, neurodegeneration

## Abstract

Age-related impairment of mitochondrial function may negatively impact energy-demanding processes such as synaptic transmission thereby triggering cognitive decline and processes of neurodegeneration. Here, we present a novel model for age-related mitochondrial impairment based on partial inhibition of cytochrome c oxidase subunit 4 (Cox4) of complex IV of the respiratory chain. miRNA-mediated knockdown of Cox4 correlated with a marked reduction in excitatory and inhibitory synaptic marker densities *in vitro* and *in vivo* as well as an impairment of neuronal network activity in primary neuronal cultures. Transcriptome analysis identified the deregulation of gene clusters, which link induced mitochondrial perturbation to impaired synaptic function and plasticity as well as processes of aging. In conclusion, the model of Cox4 deficiency reflects aspects of age-related dementia and might, therefore, serve as a novel test system for drug development.

## Introduction

A large and complex set of parameters contributes to cellular aging including genomic instability, epigenetic alterations, deregulated nutrient sensing, and impaired proteostasis (López-Otín et al., 2013). Additionally, an age-dependent decline in mitochondrial function may lead to a decrease in cellular energy supply and dysregulation of calcium homeostasis. Mitochondrial deficits are therefore expected to have a strong impact on energy-demanding processes such as synaptic transmission and might represent a contributing factor for aspects of neurodegeneration (Grimm and Eckert, 2017), e.g., synaptic dysfunction. In line with this, neurodegenerative diseases such as Alzheimer’s disease (AD) and Parkinson’s disease (PD) have been linked to mitochondrial dysfunction that might exacerbate the impact of aging. Likewise, brains from animal models for aging display a decrease in oxygen consumption as well as ATP production, both tightly linked to proper mitochondrial function (Xu et al., 2007; Hiona et al., 2010; Stefanatos and Sanz, 2018).

In the case of animal models of familial AD (FAD), that rely on inherited mutations driving the pathology, mitochondrial impairments occur already early with respect to disease progression suggesting mitochondrial physiology as an interesting therapeutic target (Hauptmann et al., 2009; Yao et al., 2009). Accordingly, a FAD model shows a decreased expression of subunit 4 of cytochrome c oxidase (Cox), also termed complex IV, decreased Cox activity and decreased mitochondrial respiration in parallel with an increase in oxidative stress (Yao et al., 2009). Importantly, activity of Cox was also shown to be reduced in patients diagnosed with MCI or AD (Mutisya et al., 1994; Parker and Parks, 1995; Bosetti et al., 2002; Cardoso et al., 2004; Valla et al., 2006; Selfridge et al., 2013).

Cox is composed of 13 subunits encoded by three mitochondrial and ten nuclear genes. The complex is the terminal component of the mitochondrial respiratory chain and serves for the transfer of electrons to oxygen. Cox4 was previously suggested to be essential for the assembly of the cytochrome c oxidase complex and therefore indispensable for proper respiration and concomitant ATP production (Li et al., 2006).

Here, we characterize a model for age-related neurodegeneration based on the downregulation of Cox4. We show that the model exhibits synaptic loss and altered neuronal network activity. Furthermore, transcriptomic profiling revealed deregulation of gene clusters for synaptic function and plasticity as well as processes of aging.

## Materials and Methods

### Construction of Lentiviral and AAV miRNA Expression Vectors

Target sequences for the miRNA-mediated knockdown of Cox4 isoform 1 were identified with the help of the Block-iT^TM^ RNAi Designer^1^. Target sequences chosen were ATAGTCTTCACTCTTCACAAC for miCox79 and TTCTTGTTGTAGTCCCACTTG for miCox474. Synthetic double-stranded polynucleotides harboring the target sequences in a sense-loop-antisense configuration were designed and ligated into pcDNA^TM^6.2-GW/miR (Thermo Fisher Scientific, Waltham, MA, USA). All constructs were validated by sequencing before site-specific Gateway^®^ recombination (Thermo Fisher Scientific, Waltham, MA, USA) was applied to transfer miRNA knockdown cassettes from newly generated pcDNA^TM^6.2-GW/miR vectors or a negative control miRNA from pcDNA^TM^6.2-GW/miR-neg control plasmid (Thermo Fisher Scientific, Waltham, MA, USA) into pLenti04C/SEW or pAAV-C1.3_mCherry_attR_WPRE. pLenti04C/SEW was based on pLenti6/V5-DEST^®^ (Thermo Fisher Scientific, Waltham, MA, USA), pAAV_C1.3_mCherry_attR_WPRE was generated from pAAV-CaMKIIa-hM3D(Gq)-mCherry (gift from Bryan Roth; Addgene plasmid #50476^2^; RRID:Addgene_50476).

For the generation of pLenti04C/SEW, CMV promoter sequences were exchanged for a functional mouse α-CaMKII promoter fragment *via* restriction cloning using ClaI and SpeI restriction sites on pLenti6/V5-DEST^®^. V5-epitope, EM7 promoter and Blasticidin resistance from pLenti6/V5-DEST^®^ were replaced by an expression cassette driving EGFP expression under the control of a rat synapsin I promoter and WPRE using XhoI (PspXI) and Bpu1102I restriction sites (Dittgen et al., 2004). For the generation of pAAV-C1.3_mCherry_attR_WPRE from pAAV-CaMKIIa-hM3D(Gq)-mCherry, hM3D sequences were first removed by BamHI digest and subsequent re-ligation of the vector. A Gateway^®^ recombination compatible cassette from pLenti6/V5-DEST^®^ containing attR1 and attR2 sites was inserted 3’ to the mCherry sequence using EcoRV and HindIII restriction sites.

### Preparation, Culture, and Treatment of Primary Neurons

Primary hippocampal or cortical neurons were prepared from E18 rat embryos of either sex by trypsin digestion and subsequent trituration of respective tissues (Goetze et al., 2003; Kriebel et al., 2011). Cells were seeded in serum-free MEM with B27 supplement (Thermo Fisher Scientific, Waltham, MA, USA) on polyethyleneimine (PEI) coated 6-Well cell culture plates (Corning), 96-well μCLEAR^®^ or SENSOPLATE microplates (Greiner Bio-One) at a density of 2.0 × 10^5^ cells/cm^2^. At 1 day *in vitro* (DIV1), the plating medium was replaced by fresh serum-free MEM with B27 supplement. Cultures were maintained at 37°C, 5% CO_2_ in either serum-free MEM with B27 supplement or in BrainPhys^TM^ Neuronal Medium containing SM1 supplement (STEMCELL Technologies) depending on the downstream application. A 50% medium change was performed every other day.

Lentiviral suspensions generated by lipofection of HEK239FT with lentiviral expression vectors and packaging plasmids pLP1, pLP2 and pLP/VSVG (Thermo Fisher Scientific, Waltham, MA, USA) were used to transduce cultured neurons at MOIs up to 10 at DIV2. After the transduction of mouse neuroblastoma Neuro-2a cells with lentiviral miRNA vectors coexpressing EGFP, biological titers (TU/ml) were determined by flow cytometry and detection of transduced, EGFP-positive cells. Each viral suspension was additionally tested by the transduction of primary rat neurons to assure quantitative transduction before *in vivo* application. AAV suspensions were produced by the Viral-Core-Facility (VCF) of the Charité—Universitätsmedizin Berlin after the provision of corresponding AAV miRNA expression vectors. The transduction of cultured neurons was carried out at DIV2 at MOI of 1 × 10^4^. SB216763 was applied at a concentration of 5 μM for 24 h.

### Stereotaxic Injection of Lentiviral Suspensions

Adult female Sprague–Dawley rats (250 g at the time of surgery; Janvier) were kept in compliance with the European Union recommendations for the care and use of laboratory animals and as approved by the responsible German regional council, respectively. For stereotaxic injection of lentiviral suspensions, animals were deeply anesthetized with 2–5% isoflurane/oxygen, followed by s.c. injection of metamizole (50 mg/kg) for intraoperative analgesia at least 30 min before the start of surgical procedures. Bilateral injections of 2.5 μl of lentiviral suspensions (2 × 10^7^ TU/ml) into the dorsal dentate gyrus (AP: −2.9 mm, ML: ±2.5 mm, DV: −4.3 mm; all coordinates relative to Bregma) were conducted using a Lab Standard^TM^ Stereotaxic Instrument (Stoelting) connected to a 701 RN Hamilton syringe (10 μl, 30 gauge, pst 4; CS-Chromatographie Service). Injection speed was set to 0.2 μl/min. To assure sufficient postoperative analgesia, 2 mg/kg meloxicam was administered by s.c. injection at the end of surgical procedures.

For fixation of brain tissue, animals were deeply anesthetized with ketamine (100 mg/kg i.p.) and xylazine (10 mg/kg i.p.) and transcardially perfused with 100 ml of PBS followed by 250 ml of freshly prepared 4% paraformaldehyde/PBS. Brain tissue was collected and additionally fixed in 4% paraformaldehyde/PBS at 4°C for 60 min.

### Quantitative RT-PCR

Total RNA of primary rat neurons quantitatively transduced on DIV2 by lentiviral or AAV suspensions was prepared at DIV14 using RNeasy Mini Kit (Qiagen) according to manufacturer’s guidelines. DNase digestion as well as cDNA synthesis were performed according to manufacturer’s protocols using RQ1 DNase (Promega) as well as M-MuLV reverse transcriptase (New England Biolabs). Quantitative real-time PCR was performed on the 7500 Fast Real-Time PCR System (Thermo Fisher Scientific, Waltham, MA, USA). The following TaqMan^®^ Gene Expression Assays (Thermo Fisher Scientific, Waltham, MA, USA) were used: GAPDH (VIC-MGB), Rn01775763_g1; Cox4 isoform 1 (FAM-MGB), Rn00665001-g1.

### Determination of Cell Viability, Induction of Apoptosis, ATP Content and Mitochondrial Membrane Potential

Cell viability and induction of apoptosis were determined using the ApoLive-Glo^TM^ Multiplex Assay (Promega) according to the manufacturer’s guidelines. ATP content was determined using the CellTiter-Glo^®^ Luminescent Cell Viability Assay (Promega) according to the manufacturer’s protocols. For both assays, primary rat neurons cultivated in 96-well μCLEAR^®^ microplates were transduced with lentiviral suspensions at DIV2 and cultivated until DIV16. Where applicable, cultures were treated with picomolar concentrations of rotenone 30 min before measurement. Fluorescence and luminescence were detected with a Pherastar microplate reader (BMG Labtech).

Changes in mitochondrial membrane potential (MMP) of rat primary neurons were assessed using JC-10 (Enzo Life Sciences, Farmingdale, NY, USA). Neuronal cultures in 96-well μCLEAR^®^ microplates were transduced with lentiviral suspensions at DIV2, cultivated until DIV14 and incubated with 4 μM JC-10 in culture medium containing 0.02% Pluronic F127 (Merck) for 20 min. JC-10 emission ratio (590 nm/525 nm) was determined with an EnVision 2102 Multilabel Reader. FCCP (Merck) was added to the cultures after JC-10 labeling before fluorescence measurements.

### Immunocytochemistry, Immunohistochemistry

Primary rat neurons cultured in 96-well SENSOPLATE microplates were fixed at DIV16 using 4% paraformaldehyde/PBS for 10 min at room temperature. After blocking and permeabilization for 30 min at room temperature in 0.2% Triton X-100/PBS containing 1× Blocking Reagent for ELISA (Merck) cells were incubated overnight at 4°C with primary antibodies diluted in blocking solution. Subsequently, cells were washed three times in PBS before fluorescently labeled secondary antibodies (1:500; Cy3/Cy5-coupled goat anti-mouse and goat anti-rabbit, respectively; Dianova) were added for 2 h at room temperature. Nuclei were stained using Hoechst Dye 33258 (1:1,000 in PBS; Merck). The following primary antibodies were used: monoclonal mouse anti-Cox4 (Cell Signaling Technology, Danvers, MA, USA, Cat. No. 11967), monoclonal mouse anti-gephyrin (Synaptic Systems, Cat. No. 147021), monoclonal mouse anti-MAP2 (Merck, Cat. No. M1406), polyclonal rabbit anti-MAP2 (Merck, Cat. No. AB5622), monoclonal mouse anti-NeuN (Merck, Cat. No. MAB377), polyclonal rabbit anti-PSD95 (Abcam, Cat. No. ab18258), monoclonal rabbit anti-PSD95 (Cell Signaling Technology, Danvers, MA, USA), polyclonal rabbit anti-VGAT (Synaptic Systems, Cat. No. 131003), monoclonal mouse anti-VGlutI (Synaptic Systems, Cat. No. 135511).

Perfusion-fixed brains were washed in PBS and cut into free-floating sections using a vibrating microtome set at 70 μm section thickness (Vibratom VT1000S; Leica Biosystems). After permeabilization for 1 h at room temperature in 0.6% Triton X-100/PBS slices were blocked in 1× Blocking Reagent for ELISA (Merck) for 1 h before being incubated with primary antibodies overnight at room temperature. Further immunohistochemical staining of brain slices followed the procedure described above for primary cells. Specimens were coverslipped on microscopic slides using Dako Fluorescent Mounting Medium (Dako).

### Image Acquisition and Analysis

Confocal fluorescence microscopy was performed using a Zeiss LSM510 Meta confocal microscope equipped with a 63× Plan-Apochromat oil immersion objective, NA 1.4 (Carl Zeiss Microscopy). Confocal image stacks of stained neuronal cultures and hippocampal slices were recorded with LSM510 software [microns per pixel (x, y, z): 0.28 μm, 0.28 μm, 0.5 μm] with excitation and emission settings kept constant during the recording of sample material from different experimental groups.

### Determination of Cox4 Immunoreactivity *in vitro*

Protein expression levels of Cox4 *in vitro* were quantified with ZEN 2 (Carl Zeiss Microscopy) in confocal images of lentivirally transduced primary neuronal cultures immunocytochemically stained against Cox4. To this end, mean gray value intensities of Cox4 immunoreactivity in 50 μm^2^ somatic regions of interest (ROI) of EGFP positive neurons were quantified with the help of ZEN’s measure function. ROIs were placed in neuronal cytoplasm in confocal z-planes which showed maximum diameters of neuronal somata.

### Determination of Cox4 Immunoreactivity *in vivo*

Quantification of Cox4 expression *in vivo* was carried out on confocal z-stacks of lentivirally transduced hippocampal tissue immunohistochemically stained for Cox4 and labeled with the nuclear dye Hoechst 33258. Image stacks were loaded into Imaris 8 (Bitplane) and 3-dimensional ROIs of EGFP positive neuronal cell somata were generated using Imaris’ surface creation functionality. Respective ROIs were used to mask EGFP fluorescent signals and to subsequently generate a new image channel representative of the somatic EGFP signal (EGFP^soma^) determined by the ROIs selected. To exclude nuclear regions from the downstream quantification of Cox4 immunoreactivity, additional ROIs for neuronal nuclei (positive for Hoechst 33258) were generated using Imaris’ surface tool and subtracted from the EGFP^soma^ image channel resulting in another image channel devoid of nuclear regions (EGFP^cytoplasm^). Three-dimensional ROIs of the EGFP^cytoplasm^ image channels generated using Imaris’ surface tool were finally used to quantify mean gray value intensities of Cox4 immunoreactivity and to generate another image channel representing Cox4 immunoreactivity located inside the cytoplasm of EGFP positive neurons.

### Determination of Neuronal Survival *in vivo*

Quantification of Draq5 positive nuclei in the granule cell layer of the dentate gyrus was carried out using Imaris’ spots creation functionality. Confocal image stacks of the hippocampal granule cell layer immunohistochemically stained for NeuN and labeled with the nuclear dye Draq5 were loaded into Imaris. Appropriate values for expected spot size were set (7 μm) and nuclei were individually detected as spots in defined ROIs [x, y, z (μm): 70, 135, 30] inside the granule cell layer. To initially investigate whether all nuclei present in the granule cell layer were of neuronal origin, the colocalization of Draq5 signals and the neuronal marker NeuN was analyzed. First, the number of Draq5 signals was determined as described above. Resulting spot structures were used to mask the NeuN channel creating a new image channel. The new image channel was then used to run a second round of spot detection detecting only NeuN positive cell nuclei (see also Supplementary Figure S5). The reliability of the method described above was confirmed by comparing the results of a manual count with those of the automated determination. Analysis of five different confocal z-stacks yielded an accuracy of the automated protocol of 98.99% [±1.36 (SEM)].

### Quantification of Clusters of Synaptic Marker Proteins

Quantification of immunocytochemically/immuno- histochemically labeled clusters of synaptic marker proteins was carried out using confocal z-stacks of either additionally MAP2 stained primary rat neuronal cultures or hippocampal tissue, both transduced with respective lentiviral suspensions. Twenty micrometer segments of proximal dendrites were defined as ROIs using Imaris’ surface creation tool and either MAP2 immunoreactivity (primary neurons) or EGFP expression (hippocampal tissue). The position of ROIs was solely based on MAP2 immunoreactivity or EGFP fluorescence to avoid any bias towards the distribution of synaptic marker protein staining. Generated ROIs were used to mask image channels of stained synaptic marker proteins allowing for the isolation of the corresponding immunoreactivity located in the previously defined ROI. The number of synaptic marker protein clusters was then determined using the Imaris’ surface creation tool, which was applied to the ROI-specific immunoreactive signals of the synaptic marker protein analyzed.

### Calcium Imaging

Cal520^®^-AM (AAT Bioquest) was originally dissolved in DMSO containing 20% (v/v) Pluronic^®^ F-127 to yield stock solutions of 10 mM. At DIV14, primary rat neurons cultivated in 96-well μCLEAR^®^ microplates and transduced with AAV suspensions on DIV2 were labeled by incubation with 1 μM Cal520^®^-AM in culture medium for 30 min. Subsequently, calcium transients, i.e., temporal changes in green fluorescence, were recorded in complete culture medium using an FDSS/μCell (Hamamatsu). Raw data was analyzed using FDSS software and Origin 2015 (OriginLab Corporation, Northampton, MA, USA).

### Western Blotting

Primary rat neurons were cultivated in 6-well plates and harvested in lysis buffer (10 mM Tris-HCL pH 7.5, 100 mM EDTA, 10 mM NaCl, 0.5% (v/v) Triton-X-100, 0.5% (w/v) Sodium deoxycholate) on ice at DIV16. Protein concentrations were determined with the Pierce^TM^ BCA Protein Assay Kit (Thermo Fisher Scientific, Waltham, MA, USA) according to manufacturer’s guidelines. Lysates were separated by SDS-PAGE and transferred to a nitrocellulose membrane followed by incubation with the following primary antibodies: monoclonal mouse anti-gephyrin (1:1,000; Synaptic Systems), monoclonal mouse anti-β-actin (1:10,000; Merck). Bound primary antibodies were detected using fluorescently labeled, mouse-specific secondary antibodies (Dianova). Corresponding fluorescent signals were acquired with a Typhoon Trio Variable Mode Imager (GE Healthcare).

### Transcriptome Sequencing

For transcriptome sequencing, primary rat neurons were lysed in RLT buffer at DIV16 and total RNA was extracted using the RNeasy Mini Kit (Qiagen). The quality and quantity of RNA were measured using an RNA 6000 Nanochip on the 2100 BioAnalyzer (Agilent Technologies). Thirty nanogram of total RNA was converted to cDNA using the Ovation RNA-Seq System V2 (Nugen Technologies) according to manufacturer’s protocols. This cDNA was used to construct libraries for next-generation sequencing using Ovation Rapid DR Multiplex System (Nugen Technologies) according to the manufacturer’s protocols. Sequencing was performed on HiSeq2500 instruments (Illumina) with paired-end sequencing, 100 bp read length, yielding an average of 75 million read pairs per sample.

### Detection of Differentially Expressed Transcripts

Sequencing data were processed with bcl2fastq (version 1.8.2, Illumina) to demultiplex sequencing reads. Sequencing adapters were trimmed using Skewer [version 0.1.116 (Jiang et al., 2014)]. The trimmed reads were mapped to the rat reference genome (RNor5, Illumina iGenomes) using STAR [version 2.3.0e_r291 (Dobin et al., 2013)] with default parameters. Transcription was quantified on Ensembl transcripts [release 75 (Flicek et al., 2014)] and differential expression computed using Cuffdiff [version 2.1.1 (Trapnell et al., 2013)] based on the pooled dispersion model and geometric library size normalization. Differentially expressed genes were identified at a significance threshold of *p* < 0.05 (FDR-corrected *p*-values) in two comparisons: (1) miCox79 vs. miCTR; and (2) miCox474 vs. miCTR. Mayday (Battke et al., 2010) was used for the heatmap generation. Venn diagrams were generated using InteractiVenn (Heberle et al., 2015).

### Statistics

Statistical analyses (unpaired *t*-test, Mann–Whitney test, multiple *t*-tests, one-way ANOVA, Kruskal–Wallis test, *post hoc*-tests where applicable) were performed with GraphPad Prism 7 (GraphPad Software). Unless stated otherwise, experiments were carried out three times. Apart from the quantifications *in vivo*, results shown are representative of one of three experiments conducted, which all replicated the respective experimental findings. For analyses *in vivo*, three rats per experimental condition, i.e., lentiviral suspension applied, were used. Error bars indicate the standard error of the mean (SEM).

All experimental procedures described were conducted according to standard biosecurity and institutional safety procedures.

## Results

### RNAi-Mediated Knockdown of Cox4 for Modeling Mitochondrial Impairment

**Figure 1:**
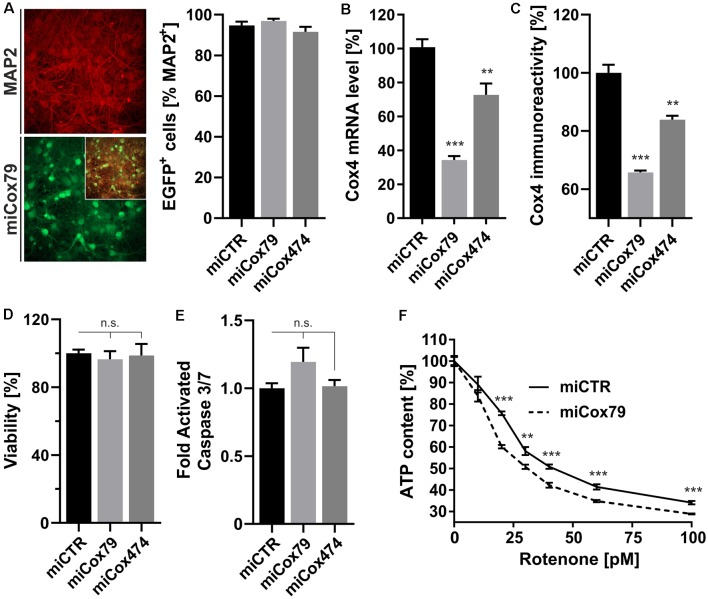
Neuron-specific knockdown of Cytochrome C Oxidase subunit 4 (Cox4). **(A)** Determination of lentiviral transduction efficiency, i.e., percentage of cells positive for both virally expressed EGFP and neuronal marker Microtubule-associated protein 2 (MAP2). Micrographs depict confocal images of a rat primary neuronal culture transduced with Lenti04CmiCox79/SEW (miCox79) and counterstained for MAP2. *n* = 10 images for all groups. **(B,C)** Knockdown of rat Cox4 expression as assessed by quantitative real-time PCR **(B)** and single-cell quantitative immunocytochemistry **(C)** in lentivirally transduced primary rat neurons *in vitro*. One-way ANOVA, Dunnett’s multiple comparisons test. ***p < 0.001; **p < 0.01. *n* = 6 wells for all groups **(B)**. Kruskal–Wallis test, Dunn’s multiple comparisons test. ***p < 0.001; **p < 0.01. *n* = 25 cells for all groups **(C)**. **(D,E)** Determination of cell viability and activated caspase 3/7 (ApoLive-Glo^TM^ Multiplex Assay) in cultures of rat primary neurons after lentiviral transduction with either miCTR, miCox79 or miCox474 expressing lentiviral vectors. One-way ANOVA, Dunnett’s multiple comparisons test. n.s., not significant. *n* = 6 wells for all groups. **(F)** Determination of ATP content in primary rat neurons transduced with miCTR or miCox79 expressing lentiviral vectors and treated with picomolar concentrations of rotenone for 30 min before measurement. Multiple *t*-tests. ***p < 0.001; **p < 0.01. *n* = 6 wells for all groups.

A decline of complex IV activity has been detected in individuals diagnosed with MCI (Mutisya et al., 1994; Cardoso et al., 2004). To generate a corresponding model of age-related mitochondrial impairment, we, therefore, chose to interfere with the expression of subunit 4 (Cox4) of cytochrome c oxidase, a subunit reported being essential for assembly and respiratory function of complex IV (Li et al., 2006). Lentiviral vectors were employed that expressed Cox4-specific miRNAs, namely miCox79 or miCox474, or a control miRNA under the control of a mouse CaMKII promoter, which restricts miRNA expression to neurons. Furthermore, the expression of EGFP under the control of a rat synapsin promoter allowed for the identification of successfully transduced cells *in vitro* and *in vivo* (Figures 1A, 4A). Knockdown efficacies of either miCox79 or miCox474 were first determined by qRT-PCR after the quantitative transduction of rat primary neurons *in vitro*. miCox79 and miCox474 significantly reduced Cox4 mRNA levels to 34% and 73% of control transduced cells (miCTR; Figure 1B). The functionality of both miCox79 and miCox474 was confirmed on the protein level utilizing quantitative immunocytochemistry using an antibody specific for Cox4 (Figure 1C).

**Figure 2:**
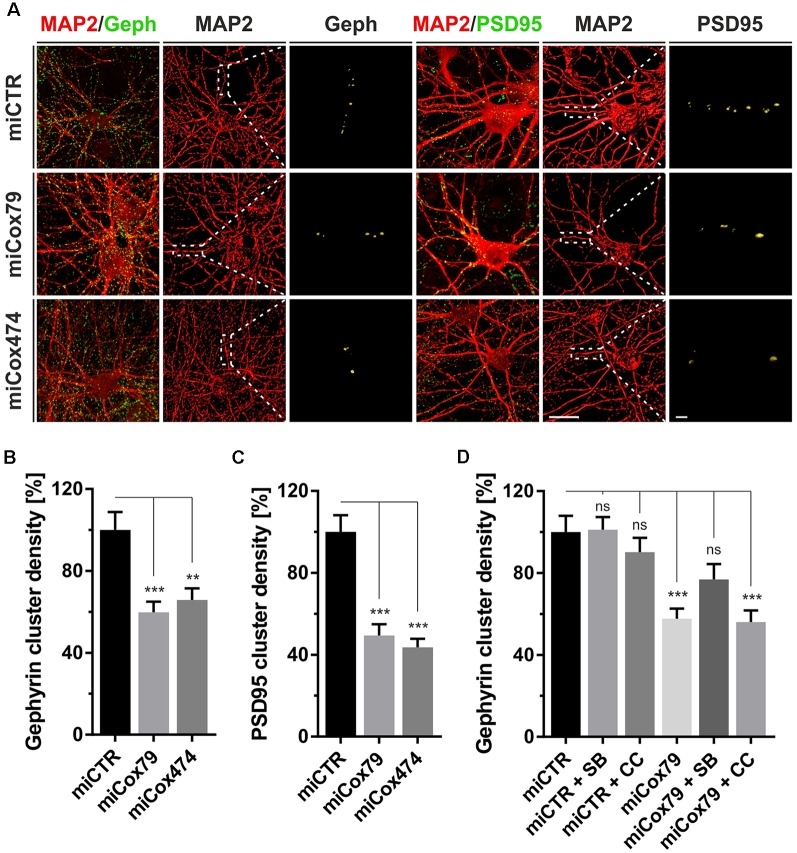
Synaptic loss *in vitro* after knockdown of Cox4. **(A)** Micrographs of cultured primary rat neurons transduced with lentiviral suspensions for the expression of either miCTR, miCox79, or miCox474 and stained for neuronal markers MAP2, Gephyrin (Geph), and PSD95, respectively. The respective left images (EGFP/Geph, EGFP/PSD95) represent maximum intensity projections (MIP) of confocal z-stacks. The remaining tiles depict neuronal cultures after surface rendering using immunofluorescent signals of marker proteins indicated. Segmentation of Gephyrin and PSD95 positive immunofluorescent signals was carried out on 20 μm segments of primary dendrites (dashed boxes). Scale bars, 20 μm, and 2 μM, respectively. **(B,C)** Quantification of Gephyrin and PSD95 cluster densities on 20 μm segments of primary dendrites of hippocampal neurons transduced as indicated. *n* = 39 dendritic segments for all groups. Kruskal–Wallis test, Dunn’s multiple comparisons test. ***p < 0.001; **p < 0.01. **(D)** Quantification of Gephyrin cluster densities on 20 μm segments of primary dendrites. Hippocampal neurons were transduced with lentiviral suspensions and treated with pharmacological inhibitors as indicated. SB, SB216763 (GSK3 inhibitor); CC, Compound C (AMPK inhibitor). Kruskal–Wallis test, Dunn’s multiple comparisons test. ***p < 0.001. ns, not significant. *n* = 40 dendritic segments for all groups.

**Figure 3:**
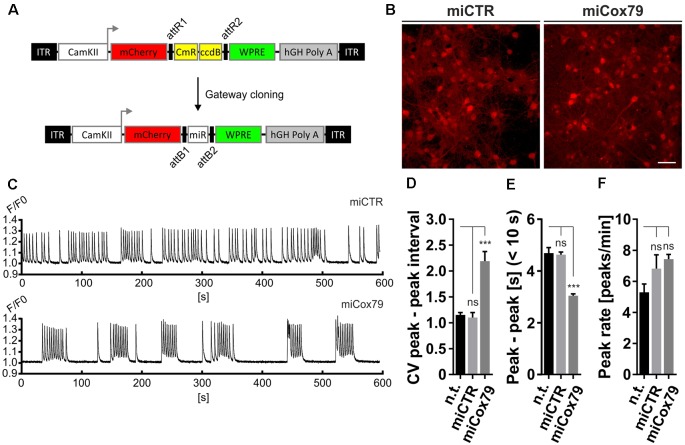
Altered network rhythmicity after knockdown of Cox4. **(A)** Structure of AAV constructs for bicistronic expression of mCherry and miRNA sequences under the control of a functional mouse α-CaMKII promoter fragment. Respective miRNA sequences are transferred by Gateway cloning. ccdB, ccdB gene; CmR, Chloramphenicol resistance; ITR, inverted terminal repeats; WPRE, Woodchuck Hepatitis Virus Posttranscriptional Regulatory Element. **(B)** Successful transduction of rat primary neurons using both AAV_C1.3_mCherry_miCTR_W (miCTR) and AAV_C1.3_mCherry_mi79_W (miCox79) viral suspensions as assessed by mCherry expression. Scale bar, 50 μm. **(C)** Recordings of intracellular calcium levels indicative of neuronal network activity after transduction of primary rat neurons with either miCTR or miCox79 expressing AAVs. Intracellular calcium transients were detected using Cal-520^®^ AM. Each graph depicts the Cal-520^®^ AM intensity (F/F0 ratio) recorded from one well of a 96-Well plate using Hamamatsu’s FDSS/μCELL. **(D–F)** Quantification of peak-to-peak interval coefficient of variation (CV), peak-to-peak interval [s] filtered for intervals <10 s, and average peak rate (peaks/min) from recorded cultures transduced as indicated. One-way ANOVA, Dunnett’s multiple comparisons test, *n* = 6 wells for all groups in **(D,F)**. **(E)** Kruskal–Wallis test, Dunn’s multiple comparisons test, *n* = 168 (n.t.), 367 (miCTR), and 321 (miCox79) from six wells each. ***p < 0.001. ns, not significant; n.t., not treated.

**Figure 4:**
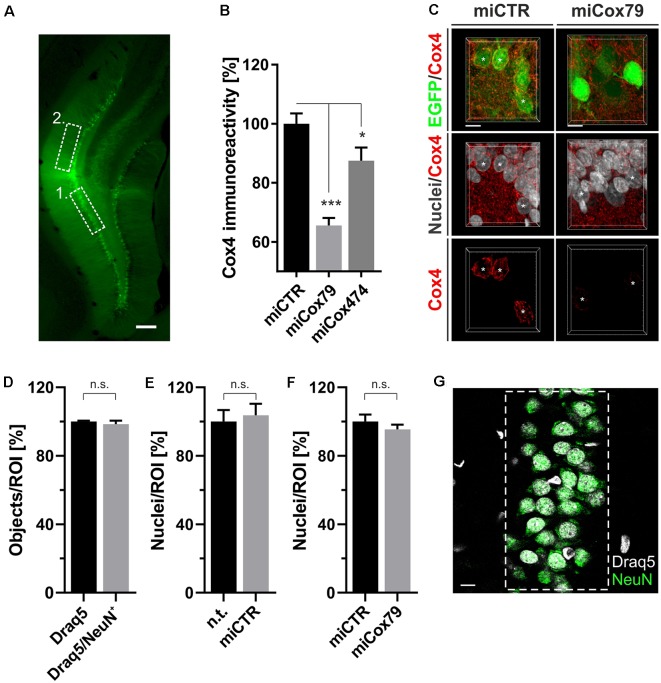
**(A)** Successful transduction of granule cells of the dentate gyrus *in vivo* as shown by EGFP expression after injection of Lenti04CmiCTR/SEW. Dashed boxes indicate the areas of analysis for 1. Determination of Cox4 expression **(B,C)** and neuronal survival **(D–G)**, i.e., the granule cell layer, and 2. assessment of synaptic connectivity (Figure 5), i.e., the molecular layer of the suprapyramidal blade. Scale bar, 200 μm. **(B)** Knockdown of rat Cox4 expression as assessed by single-cell quantitative immunohistochemistry in hippocampal granule cells after stereotaxic injection of respective lentiviral suspensions *in vivo*. One-way ANOVA, Dunnett’s multiple comparisons test. ***p < 0.001; *p < 0.05. *n* = 40 cells for miCTR, *n* = 42 cells for miCox79, and *n* = 25 cells for miCox474. **(C)** MIP of confocal image stacks from hippocampal granule cells after transduction with miCTR or miCox79 expressing lentiviral vectors. Asterisks mark successfully transduced granule cells (EGFP positive) selected for quantification of Cox4 immunoreactivity. Scale bars, 10 μm. **(D)** High percentage of neuronal cells (98%) in the granule cell layer of the dentate gyrus of the rat, as shown by the NeuN labeling of Draq5 stained nuclei. Unpaired *t*-test. n.s., not significant. *n* = 3 animals. Average number of nuclei detected per animal (12 confocal stacks): 653 (Draq5), 629 (Draq5/NeuN). Neuronal cell numbers are not affected under either control **(E)** or Cox4 knockdown conditions **(F)**. Unpaired *t*-test. *n* = 3 animals for both groups in **(E,F)**. n.s., not significant. Average number of nuclei detected per animal (12 confocal stacks) in **(E)**: 648 [n.t. (not treated)], 672 (miCTR). Average number of nuclei detected per animal (15 confocal stacks) in **(F)**: 857 (miCTR), 820 (miCox79). **(G)** Dashed box indicates the region of interest (ROI) delineating the granule cell layer which was used for quantifications in **(D–F)**. Scale bar, 10 μm.

A salient problem of interference with components of the mitochondrial respiratory chain is a potential effect on general cellular viability. Decreased viability may systemically and unspecifically affect cell survival and thereby preclude the analysis of drugs designed to modulate cellular physiology, e.g., intracellular signaling pathways. An analysis of cell viability and induction of apoptosis (Figures 1D,E), overall ATP content and MMP (Supplementary Figures S1A,B) in cultures of rat primary neurons revealed no significant differences between Cox4 knockdown and control. However, ATP content was significantly decreased by up to 15% after coincubation with a minimum of 20 pM of the complex I inhibitor rotenone (Figure 1F). In conclusion, our observations argue for a mild impairment of mitochondrial function after partial Cox4 knockdown, which was detectable after additional treatment with rotenone as a second stressor.

### Cox4 Knockdown Impairs Synaptic Connectivity

Synaptic loss is a hallmark of neurodegenerative diseases and is mechanistically linked to early symptoms like MCI in the case of AD. We, therefore, determined cluster densities of synaptic marker proteins Gephyrin and PSD95 on dendritic segments of rat primary neurons after knockdown of Cox4. Quantitative immunocytochemistry revealed that miRNAs for Cox4 knockdown decreased Gephyrin cluster densities, indicative for GABAergic postsynaptic components, to 60% and 66%, respectively. PSD95 cluster densities, indicative for glutamatergic postsynaptic components, were decreased to 49% and 44% (Figures 2A–C). The significant reduction of synaptic marker densities by two independent miRNAs targeting Cox4 excluded potential off-target effects.

To additionally confirm that the observed reduction in synaptic marker densities is not due to an unspecific, toxic effect of mitochondrial impairment caused by knockdown of Cox4, the efficacy of drugs suggested to modulate clustering of Gephyrin was tested. We chose the GSK3β antagonist SB216763 since GSK3β is known to negatively regulate Gephyrin clustering (Wuchter et al., 2012; Tyagarajan et al., 2013). Likewise, the AMPK inhibitor compound c was applied to potentially upregulate mTOR activity. AMPK, which is a negative regulator of mTOR, the latter being required for Gephyrin clustering, becomes activated upon mitochondrial deficits (Wuchter et al., 2012; Beuter et al., 2016). Neither SB216763 nor compound c significantly modulated Gephyrin cluster densities in rat primary neurons expressing miCTR. By contrast, inhibition of GSK3β by SB216763 significantly rescued the effect of Cox4 knockdown on Gephyrin cluster density number, while compound c remained ineffective (Figure 2D, Supplementary Figure S2).

The observed reduction in Gephyrin clustering might be a consequence of reduced Gephyrin expression and/or enhanced cleavage by the calcium-dependent protease calpain after the Cox4 knockdown (Tyagarajan et al., 2013). However, Western Blot analysis revealed that Gephyrin protein levels remained unaffected after knockdown of Cox4 (Supplementary Figure S3).

In summary, partial knockdown of Cox4 expression *in vitro* reduced the densities of excitatory as well as inhibitory synaptic markers. Furthermore, the observed decrease in Gephyrin cluster density is explained most likely by dissociation of Gephyrin scaffolds from postsynapses rather than a depletion of Gephyrin molecules.

The observed loss of essential synaptic components might result in neuronal network perturbations at the functional level. Therefore, calcium transients were measured in rat primary neurons loaded with the synthetic calcium sensor Cal520^®^-AM. miCox79 and miCTR sequences were expressed after transduction *via* AAV vectors (Figure 3A). Coexpression of mCherry (transduction control) and functionality of miCox79 was assured using fluorescence microscopy and qRT-PCR analysis of transduced cultures (Figure 3B, Supplementary Figure S4). At 14 DIV, control cultures displayed robust network activity as shown by rhythmic and synchronized peaks (Figure 3C, upper panel). In the presence of Cox4 knockdown (Figure 3C, lower panel), the rhythmicity of network activity decreased while network synchronicity was retained. Accordingly, calculation of the coefficient of variation (CV) of peak-to-peak interval revealed a significant increase in the case of Cox4 knockdown, while no difference was found between untransduced and miCTR transduced cultures (Figure 3D). Determination of peak rates (peaks/minutes) showed no significant differences between untreated and miCTR or miCox79 expressing cultures (Figure 3F), pointing towards high peak frequencies in episodes of elevated network activity in the case of Cox4 knockdown (Figure 3C, lower panel). Indeed, quantification of peak-to-peak intervals filtered for intervals shorter than 10 s to address episodes of elevated network activity revealed a highly significant decrease in the duration of peak-to-peak intervals for miCox79 expressing cultures when compared to either untreated or control transduced cultures (Figure 3E). In summary, the decrease in synaptic density after knockdown of Cox4 described above coincides with decreased network rhythmicity.

### Knockdown of Cox4 Induces Synaptic Loss *in vivo*

**Figure 5:**
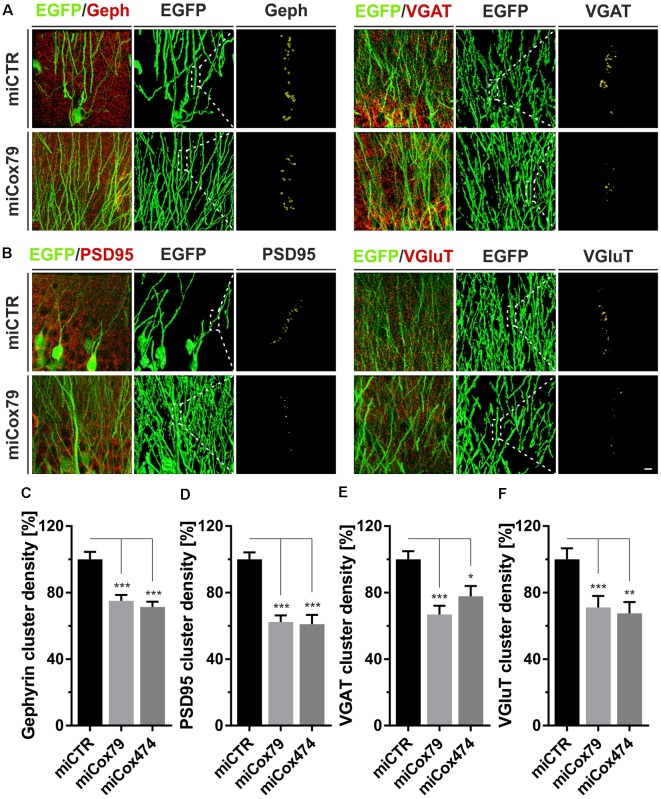
Synaptic loss *in vivo* after knockdown of Cox4. Micrographs of hippocampal granule neurons transduced with lentivirus expressing EGFP and miCTR or miCox79 and stained for synaptic markers Gephyrin (Geph) and VGAT **(A)** or PSD95 and VGluT **(B)**. The respective left images (EGFP/Geph, EGFP/VGAT, EGFP/PSD95, EGFP/VGluT) represent MIP of confocal z-stacks. The remaining tiles depict hippocampal granule neurons after surface rendering using immunofluorescent signals of the marker proteins indicated. Segmentation of synaptic markers was carried out on 20 μm dendritic segments (dashed boxes). Scale bar, 2 μm. **(C–F)** Quantification of Gephyrin, PSD95, VGAT, and VGluT cluster densities on 20 μm segments of primary dendrites of hippocampal granule neurons transduced as indicated. Shown are combined results from three (miCTR, miCox79) and two animals (miCox474), respectively. Kruskal–Wallis test, Dunn’s multiple comparisons test. ***p < 0.001; **p < 0.01; *p < 0.05. **(C)** *n* = 150 for miCTR and miCox79, *n* = 145 for miCox474; **(D–F)** *n* = 90 for miCTR and miCox79, *n* = 60 for miCox474.

Employing stereotaxic injections of lentiviral suspensions into the dentate gyrus of the hippocampus of adult female rats we wanted to further examine potential effects of a knockdown of Cox4 with respect to neuronal survival or synaptic connectivity *in vivo*. Successful transduction of hippocampal neurons and subsequent neuronal expression of EGFP led to prominent labeling of the dentate gyrus which allowed for a clear identification of its granule cell layer and molecular layer (Figure 4A). We detected a spread of viral transduction, i.e., EGFP-positive neurons, 1–2 mm in the anterior-posterior direction. Using quantitative immunohistochemistry, the successful knockdown of Cox4 was confirmed in somata of transduced granule cells (66% and 87% of residual Cox4 immunoreactivity for miCox79 and mi474, respectively; Figures 4B,C). Of note and underlining the findings *in vitro*, partial Cox4 knockdown did not show any detectable impact on neuronal survival *in vivo* 4 weeks after lentiviral transduction (Figures 4D–G) as assessed by the number of neuronal somata in the granule cell layer. We further assessed whether the phenotype of reduced synaptic connectivity could be reproduced *in vivo*. To this end, brain slices obtained from stereotaxically injected rats were prepared for immunohistochemistry 4 weeks after surgery and stained for inhibitory as well as excitatory pre- and postsynaptic markers. Z-stacks of the dendritic trees of hippocampal granule neurons extending into the molecular layer of the dentate gyrus were recorded by confocal laser scan microscopy and submitted to 3D reconstruction and quantification of clusters of synaptic marker proteins (Figure 5). Concurrent to our findings in primary cultures *in vitro*, both miCox79 and miCox474 significantly reduced the densities of postsynaptic Gephyrin (75% and 71%) and PSD95 clusters (62% and 61%) compared to controls (Figures 5C,D). Moreover, presynaptic clusters of VGAT (67% and 78%) and VGluT (71% and 68%) were also diminished in number (Figures 5E,F). Thus, pre- and post-synaptic markers of excitatory and inhibitory synapses were significantly reduced after Cox4 knockdown *in vivo*. In conclusion, our *in vivo* findings strongly corroborate the *in vitro* phenotype of impaired synaptic connectivity and underscore the validity of the *in vitro* test system.

### Transcriptome Analysis

**Figure 6:**
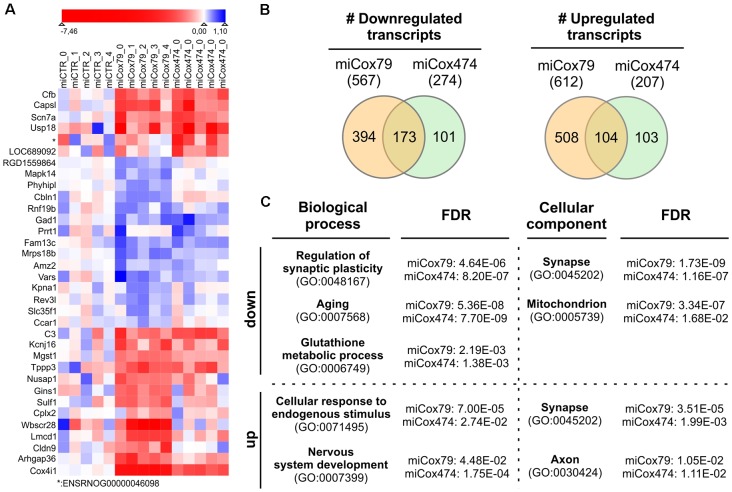
Transcriptomic alterations after knockdown of Cox4. **(A)** Heatmap of top differentially expressed genes. The top 10 downregulated and top 10 upregulated genes (by fold change between conditions) were selected both for miCox79 and miCox474. Due to overlap in the lists between the miCox79 and miCox474 conditions, 34 genes are included in the plot. Individual replicates are shown for each condition, log2 foldchange normalized to the average of the expression in all miCTR replicates is shown on a sigmoid red-blue gradient. Genes are sorted by hierarchical clustering on normalized log2 fold changes (euclidean distance). *no gene symbol associated. **(B)** Venn diagrams showing the distribution of differentially expressed transcripts following miCox79 or miCox474 treatment compared to control (miCTR). **(C)** PANTHER gene ontology (GO) analysis (statistical overrepresentation test) based on mRNAs significantly deregulated after the Cox4 knockdown. Selected GO clusters from annotation data set “biological process” and “cellular component” are shown, that were identified in both miCox79 and miCox474 datasets [Fisher’s exact test, false discovery rate (FDR) calculation]. For a complete list of GO clusters identified for miCox79 and miCox474 see Supplementary Table S1. down/up, GO clusters identified based on a list of downregulated and upregulated transcripts, respectively.

We analyzed the transcriptomes of lentivirally transduced primary neuronal cultures to further assess phenotypic changes after the Cox4 knockdown. As compared to controls (miCTR), miCox79 and miCox474 significantly induced upregulation of 612 and 207 annotated genes, respectively. 567 (miCox79) and 274 genes (miCox474) were found to be significantly downregulated (Figure 6B, Supplementary Table S1). Of note, 173 downregulated and 104 upregulated transcripts were independently identified in both miCox79 and miCox474 samples. This already accounted for 63% of all downregulated and 50% of all upregulated transcripts found in miCox474. In addition, plotting of the top 10 downregulated and top 10 upregulated genes for miCox79 and miCox474 revealed clear segregation from transcriptomic signatures of miCTR treated samples (Figure 6A). As expected, Cox4 expression was downregulated after the expression of both miCox79 or miCox474. Moreover, we also identified further deregulated genes related to complex IV after expression of miCox79, the miRNA with higher efficacy over miCox474 (downregulated: Cox14, UniProtKB=Q5XFV8); upregulated: Cox6A2, UniProtKB=P10817; Cox11, UniProtKB=M0RE03; Cox18, UniProtKB=D4A568).

Deregulated genes were assigned to gene ontology (GO) clusters with the help of the PANTHER classification tool^3^ [(Mi et al., 2013); annotation data sets: biological process complete, cellular component complete; GO Ontology database released 2020-01-03]. GO clusters obtained (FDR < 0.05; Supplementary Table S1) were aligned between miCox79 and miCox474 treated samples to identify clusters significantly and independently enriched after application of both miRNAs pointing towards a specific effect of Cox4 knockdown. 456 common GO clusters for biological processes emerged using either (miCox79, miCox474) list of downregulated genes, 71 GO clusters were identified for cellular components. These numbers made up 77% and 92% of all clusters identified for miCox474, respectively. In comparison, only three common GO clusters for biological processes and nine common GO clusters for cellular components were found based on upregulated genes (60% and 90% of all clusters identified for miCox474, respectively).

GO clusters found after assignment of downregulated genes were linked to synaptic function, cellular responses to oxidative stress, as well as aging. This is in line with our working hypothesis that an age-related decline of mitochondrial function is linked to synaptic impairments that are faithfully reproduced in our model (Figure 6C). Findings in the annotation data set biological process complete was paralleled by the GO cluster “synapse” (GO:0045202) for cellular components. Besides, genes belonging to the GO cluster “mitochondrion” (GO:0005739) were identified as significantly overrepresented in both miCox79 and miCox474 lists of downregulated genes. Concerning upregulated genes, the appearance of the cluster “synapse” (GO:0045202) underlines a dysregulation of genes involved in synaptic functioning as GO clusters for synaptic functions and cellular compartment were also identified in lists of downregulated genes.

## Discussion

Here, we present a novel model for age-related neurodegeneration based on mitochondrial impairment through partial inhibition of Cox4 expression. The model displays construct validity by interfering with complex IV known to be impaired in AD. Face validity is suggested by reduced synapse numbers and transcriptomic profiles that highlight aspects of age-related neuronal dysfunction. Thus, the Cox4 knockdown model appears to be a useful tool for phenotypic drug testing in the context of neuronal malfunction caused by mitochondrial impairment.

Aging is linked to mitochondrial dysfunction and concomitant cognitive decline (Trifunovic and Larsson, 2008). Therefore, interference with mitochondrial function and synaptic stability appears to be a reasonable starting point for the development of a new model for age-related dementia. Reduced synaptic connectivity in conjunction with a loss in spatial memory is the most salient symptom associated with dementia in general and AD (Lithfous et al., 2013; Spires-Jones and Hyman, 2014). Further, the course of AD is characterized by a loss of synaptic plasticity and connectivity before cell death occurs (Terry et al., 1991; Coleman and Yao, 2003). Our Cox4 knockdown model shows reduction of synaptic markers in conjunction with altered neuronal network rhythmicity *in vitro* suggesting a representation of impaired synaptic connectivity as observed in neurodegenerative phenotypes. Direct measurements of mitochondrial function and integrity like ATP content or MMP did not show major abnormalities under basic load. However, co-treatment of neuronal cultures with picomolar concentrations of the complex I inhibitor rotenone revealed reduced levels of ATP in the presence of Cox4 knockdown. Synaptic transmission is considered a cellular process with a particularly high-energy demand. Failure of mitochondria to respond to peaks of energy demand because of a diminished spare respiratory capacity might lead to synaptic malfunction and subsequent synaptic loss as observed in our model *in vitro* as well as *in vivo*. Our *in vitro* model is likely to reflect pathogenic phenotypes only partially due to an early developmental stage of the neurons cultured as well as an unknown absolute and spatial representation of interneurons. However, results obtained using calcium imaging document alterations in synaptic transmission, i.e., overall network rhythmicity, after synaptic loss. At his stage, however, it is unclear, whether this parameter is predictive for the action of drugs.

In a previous report, a similar strategy to reduce complex IV activity was applied (Diaz et al., 2012). The Cox10 subunit of cytochrome c oxidase was deleted in conditional knockout mice after CRE expression under the control of the CaMKII promoter. These mice showed reduced survival starting at month 4 after birth and died between months 8 and 12 probably due to cortical atrophy. Accordingly, Cox10 interference was considered as a model for encephalopathy and was not applicable as a model for age-related dementia. Our model based on partial Cox4 downregulation in mature, tightly integrated neurons appeared to be less harmful since no loss of transduced neurons was observed *in vivo* throughout the investigation. Possible effects of Cox4 knockdown at later stages than tested in this report would have to be determined in subsequent studies. Likewise, an application of Cox10 interference for the development of *in vitro* test systems remains to be assessed.

Transcriptomic analysis disclosed changes in gene expression after the Cox4 knockdown that nicely correlated with our findings regarding impaired synaptic connectivity *in vitro* and *in vivo*. Deregulated transcripts identified in both miCox79 and miCox474 groups comprised important players of synaptic plasticity such as the transcription factor Egr1, the motor protein Myo6 enriched at the postsynaptic density and the serine proteinase tissue-type plasminogen activator (Plat). For Egr1, decreased expression has been reported in aged animals and models of AD correlating with associated deficits in synaptic plasticity (Koldamova et al., 2014; Penner et al., 2016). Also, analysis of post-mortem brain tissue from non-demented controls and individuals with AD revealed changes in Egr1 expression corresponding with disease progression (Hu et al., 2019). Interference with Myo6 function, necessary for proper postsynaptic AMPA receptor trafficking, was shown to reduce synaptic numbers and impair AMPA receptor recruitment to postsynaptic sites (Wu et al., 2002; Osterweil et al., 2005; Nash et al., 2010). Likewise, a role for the stabilization of postsynaptic AMPA receptors has been assigned to Plat and the tPA-plasmin system (Diaz et al., 2019), the activity of which is decreased in aged individuals and existing models of AD (Ledesma et al., 2000; Melchor et al., 2003).

Besides further emphasizing our model’s phenotype of altered synaptic connectivity, clusters of deregulated genes also shared additional similarities with gene expression patterns observed in AD. These comprised key players of the glutathione metabolic process, e.g., members of the Gst-superfamily, which have been reported to show decreased activity also in AD possibly leading to increased oxidative stress, a process that precedes AD hallmarks like the formation of senile plaques (Lovell et al., 1998; Ansari and Scheff, 2010). In this regard, further studies would be required to determine the degree of cellular oxidative stress after the Cox4 knockdown.

Since, our model did not display neuronal loss and showed responsiveness to pharmacological treatment as exemplified by inhibition of GSK3β, phenotypes observed are unlikely to be the consequence of decreased general cellular viability. We, therefore, propose partial knockdown of Cox4 as a novel strategy to model early phases of age-related neurodegeneration characterized by impaired synaptic connectivity.

## Data Availability Statement

The transcriptomic data generated for this study can be found in the NIH Sequence Read Archive (SRA; https://www.ncbi.nlm.nih.gov/sra/PRJNA591113).

## Ethics Statement

The animal study was reviewed and approved by Regierungspräsidium Tübingen, Konrad-Adenauer-Str. 20, 72072 Tübingen.

## Author Contributions

MK constructed lentiviral and AAV expression vectors, determined corresponding knockdown efficacies, performed ATP and MMP (JC-10) quantifications, calcium measurements, and PANTHER GO analyses and contributed to manuscript writing. JE performed stereotaxic injections, determination of neuronal numbers *in vivo*, analyses of synaptic marker densities *in vitro* and *in vivo*, Western Blot experiments, and ATP quantifications. FB and SG conducted transcriptome sequencing and detection of differentially expressed transcripts. HV wrote the manuscript and contributed to the study design. All authors contributed to manuscript revision, read, and approved the submitted version.

## Conflict of Interest

FB and SG were employed by the company CeGaT GmbH. The remaining authors declare that the research was conducted in the absence of any commercial or financial relationships that could be construed as a potential conflict of interest.

## Footnotes

^1^https://rnaidesigner.invitrogen.com/rnaiexpress/

^2^http://n2t.net/addgene:50476

^3^http://www.pantherdb.org
