# Supplementary material for: Interference With Complex IV as a Model of Age-Related Decline in Synaptic Connectivity
Source: Front Mol Neurosci. 2020 Mar 24;13:43. doi: 10.3389/fnmol.2020.00043 (PMC7105595; doi:10.3389/fnmol.2020.00043)
Supplement: Supplementary file 1 [file Data_Sheet_1.pdf]

# *Supplementary Material*

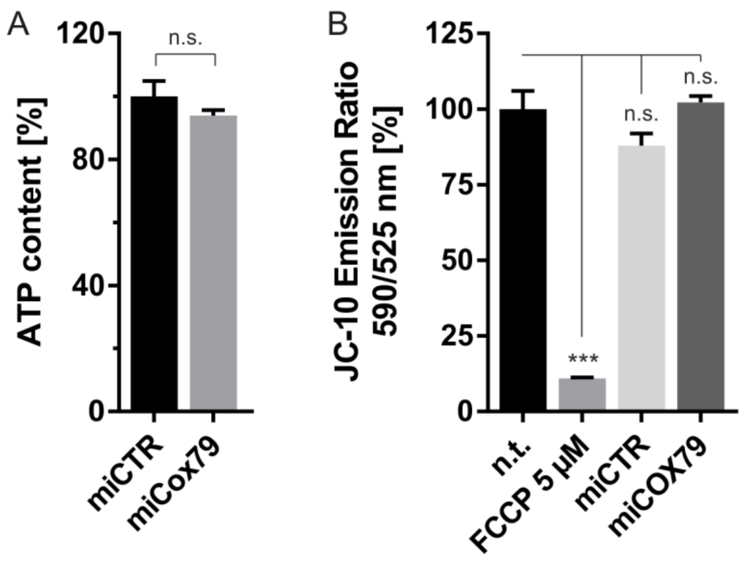


**Supplementary Figure S1.** ATP content and mitochondrial membrane potential after knockdown of Cox4. **(A)** Determination of ATP content (CellTiter-Glo® Luminescent Cell Viability Assay) in cultures of rat primary neurons after lentiviral transduction with either miCTR or miCox79 expressing lentiviral vectors. Unpaired t test with Welch's correction. n = 6 wells for both groups. n.s., not significant. **(B)** Determination of JC-10 emission ratio (590/525 nm) in primary rat neurons transduced with miCTR or miCox79 expressing lentiviral vectors. Treatment with the mitochondrial oxidative phosphorylation uncoupler FCCP served as a control. n.t., not treated. One-way ANOVA, Dunnett's multiple comparisons test. ***, $p < 0.001$. n.s., not significant. n = 6 wells for all groups.

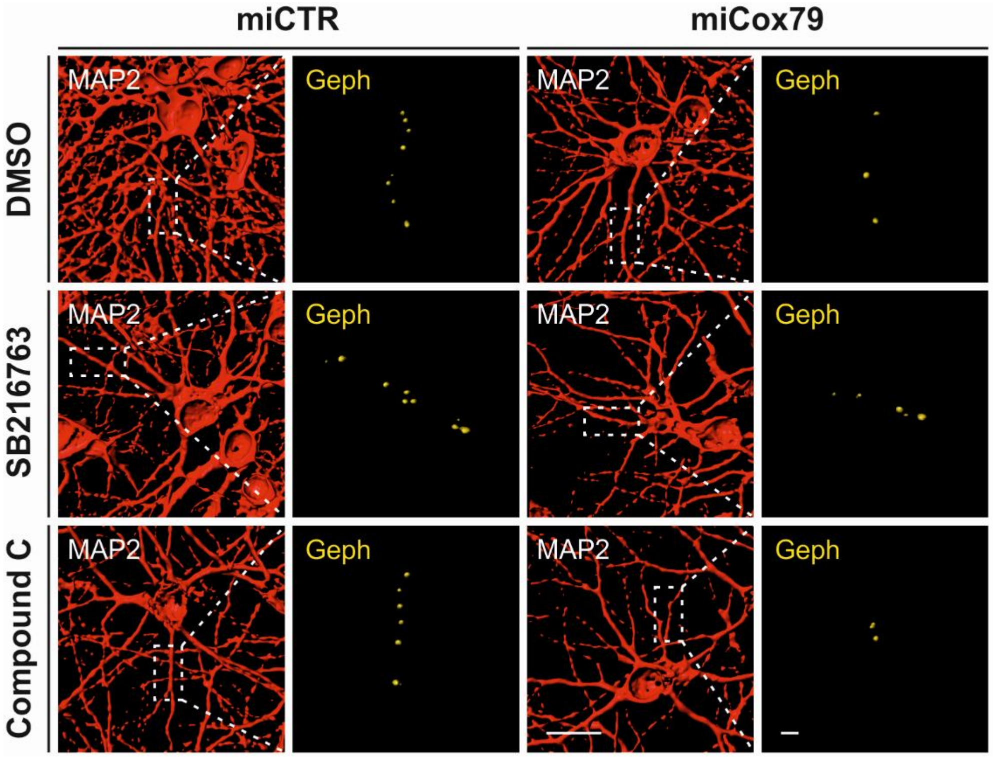


**Supplementary Figure S2.** Effects of compound treatment on Gephyrin cluster densities after knockdown of Cox4 *in vitro*. Micrographs of cultured primary rat neurons treated as indicated and stained for neuronal markers MAP2 and Gephyrin. Images depict neuronal cultures after surface rendering using immunofluorescent signals. For quantifications, see Fig. 2D. Scale bars, 20 µm and 2 µM, respectively.

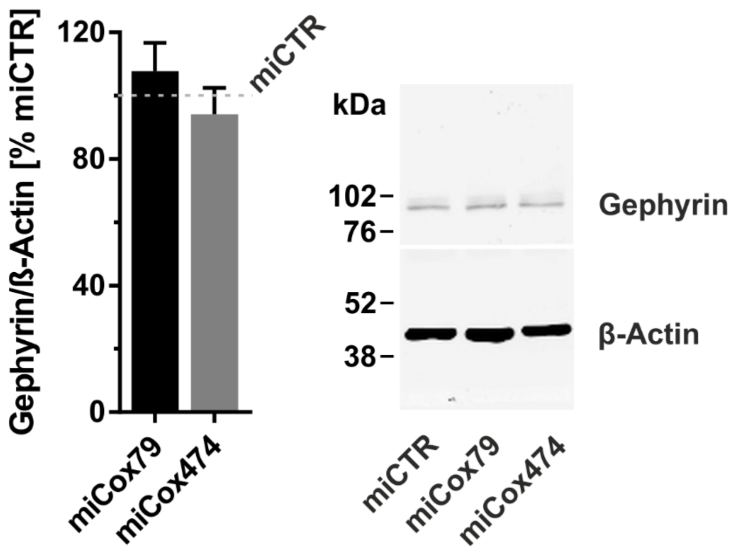


**Supplementary Figure S3.** Gephyrin expression levels remain unaltered after knockdown of Cox4. Western Blot analysis of Gephyrin protein levels in lysates from rat primary neurons after transduction with miCTR, miCox79 or miCox474 expressing lentiviral vectors. n = 3 for both groups. Shown are combined results from three independent densitometric analyses (normalized to ß-Actin) with miCTR set to 100 % in each single comparison.

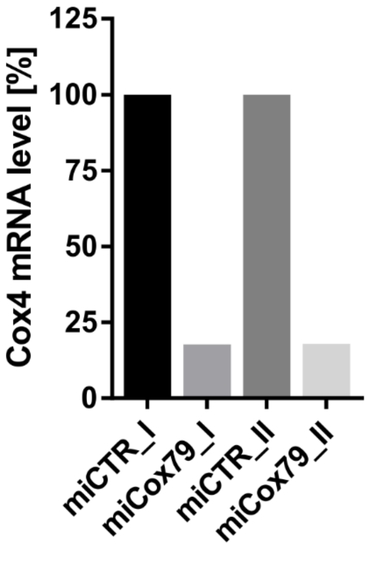


**Supplementary Figure S4.** Knockdown of Cox4 by recombinant AAV. Knockdown of rat Cox4 expression as assessed by quantitative real-time PCR in AAV transduced primary rat neurons *in vitr*o. Shown are the results from two independent transductions and quantifications termed I and II, respectively.

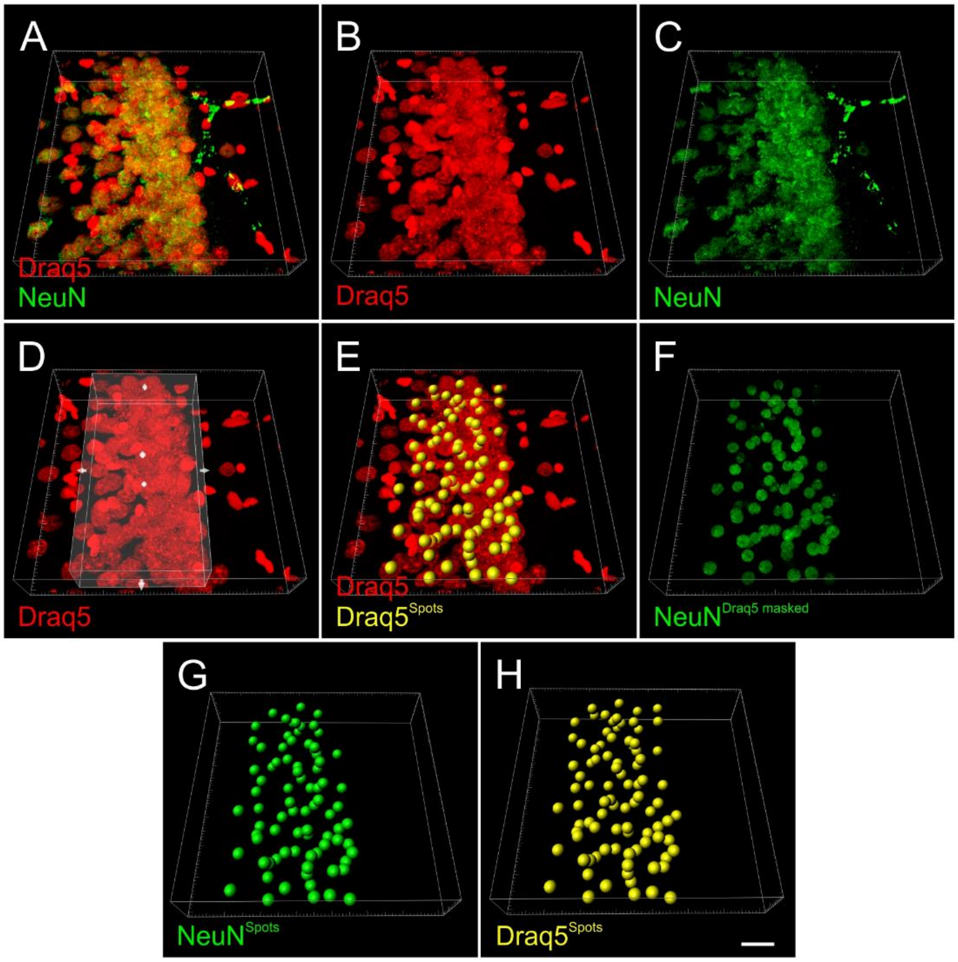


**Supplementary Figure S5.** Detection of neuronal nuclei in the granule cell layer of the dentate gyrus. **(A – C)** Maximum intensity projection of a portion of the rat dentate gyrus suprapyramidal blade stained for NeuN and labeled with the nuclear dye Draq5. **(D)** Region of interest (ROI, inner white box) used during Imaris' spots detection routine to detect Draq5-positive cell nuclei in the granule cell layer **(E)**. **(F)** Masked NeuN image channel generated by applying the Draq5-positive spots generated in (E). **(G)** NeuN-positive spots (neuronal nuclei inside the ROI shown in (D)) generated using the masked channel shown in (F). **(H)** Draq5-positive spots (cell nuclei inside the ROI shown in (D)). Scale bar, 20 µm.

**Supplementary Table S1.** Lists of genes significantly deregulated compared to control [miCTR; $p < 0.05$ (FDR-corrected p-values)] after expression of miCox79 (see tabs miCox79_down, miCox79_up) or miCox474 (see tabs miCox474_down, miCox474_up) as determined by transcriptome sequencing. Gene ontology clusters for biological processes (BP) and cellular components (CC) identified based on the lists of deregulated genes (Analysis Type: PANTHER Overrepresentation Test; FDR $p < 0.05$) are given in the tabs GO_BP_miCox79_down, GO_BP_miCox79_up, GO_BP_miCox474_down, GO_BP_miCox474_up,

GO_CC_miCox79_down, GO_CC_miCox79_up, GO_CC_miCox474_down, and GO_CC_miCox474_up, respectively.
